# Possible flat band bending of the Bi_1.5_Sb_0.5_Te_1.7_Se_1.3_ crystal cleaved in an ambient air probed by terahertz emission spectroscopy

**DOI:** 10.1038/srep36343

**Published:** 2016-11-02

**Authors:** Soon Hee Park, Sun Young Hamh, Joonbum Park, Jun Sung Kim, Jong Seok Lee

**Affiliations:** 1 Department of Physics and Photon Science, Gwangju Institute of Science and Technology, Gwangju 61005, Republic of Korea; 2 Department of Physics, Pohang University of Science and Technology, Pohang 37673, Republic of Korea

## Abstract

We investigate an evolution of the surface electronic state of the Bi_1.5_Sb_0.5_Te_1.7_Se_1.3_ single crystal, which is one of the most bulk insulating topological insulators, by examining terahertz light emitted from the sample surface upon the illumination of the near-infrared femtosecond laser pulses. We find that the surface state with a flat band bending can appear in the course of the natural maturation process of the surface state in an ambient air. Furthermore, we demonstrate that the evolution of the surface electronic state can be accelerated, decelerated, or even stopped by controlling environmental conditions to contain different amount of H_2_O, in particular.

A surface state of topological insulators (TIs) is of great importance as it provides a spin-momentum-locked Dirac dispersion which can be exploited for the spintronic applications in forms of p-n junction^1^, topological-insulator-superconductor heterostructure^2^, and even spin-dependent thermometric device^3^. Also, it was recently demonstrated that the spin injection from the topological insulator enables the magnetization switching in the adjacent magnetic layer through the spin-orbit torque^4^,^5^. However, those application exploiting peculiar topological surface states are often hindered by contributions from the bulk free carriers which mask or overwhelm the charge transport of surface carriers. Even for the sample having little bulk carriers, it is not straightforward to design the thin-film devices including a proper choice of the adjacent capping or substrate layer, for example, because of an additional charge accumulation or depletion at the TI surfaces which sometimes occurs in unexpected and uncontrollable ways^6^,^7^.

Several surface sensitive techniques have been employed to investigate TI surface states. In particular, its evolution as a function of time after the sample cleavage has been revealed by angle-resolved photoemission spectroscopy^8^,^9^, scanning tunneling spectroscopy^10^, Hall measurement^11^, and so on. Terahertz (THz) emission spectroscopy also has proven to be another important surface-sensitive technique^12^. THz light can be generated near the sample surface upon the illumination of laser pulses, and Luo *et al*. demonstrated that THz emission amplitude observed for doped Bi_2_Se_3_ depends strongly on the charge carrier density which they attributed to the contribution of Dirac fermions together with bulk carriers^13^. Also, Zhu *et al*. examined the time-dependent changes of the THz emission responses from Bi_2_Se_3_ after the sample cleavage, and demonstrated that the THz emission amplitude is changed sensitively upon the time-dependent evolution of the surface space-charge region^14^.

In this work, we investigate the evolution of the surface electronic state of the Bi_1.5_Sb_0.5_Te_1.7_Se_1.3_ (BSTS), which is one of the most bulk insulating topological insulators^15^, by exploiting THz emission technique. We cleaved the sample in various environmental conditions at room temperature, and found strong variations in the amplitude and phase of emitted THz wave as a function of time after the sample cleavage. In particular, when the sample is cleaved in an ambient air, THz emission responses exhibit non-monotonic time-dependent variations with a clear minimum at a few hours after the cleavage which we attribute to the signature of the possible flat band bending. Interestingly, the surface state evolution of the BSTS sample exhibits a remarkable controllability by adjusting the atmospheric condition; we could accelerate, decelerate, and even stop the evolution of the surface electronic states. We discuss the maturation process of the surface electronic state in the ambient air in terms of the Se vacancy formation, chemical adsorption and reaction of O_2_ and H_2_O molecules.

## Results and Discussion

### THz emission mechanism

**Figure 1:**
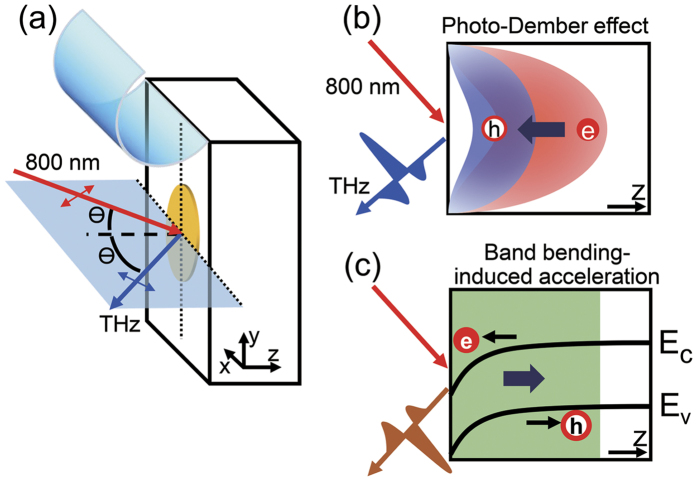
(**a**) Schematic diagram showing THz emission from the sample surface upon the illumination of near-infrared laser pulses. Incident laser and outgoing THz light are p-polarized and propagate along 45 degrees away from the surface normal direction. The blue hemi-cylinder means the cleaved layer of the BSTS crystal. (**b**,**c**) Cartoons describing two THz generation mechanisms, i.e., photo-Dember effect and band bending-induced charge acceleration, respectively. In both cases, thick arrow indicates a transient electric dipole moment formed by displacement of photo-induced electrons and holes, and its direction determines the polarity of the emitted THz wave.

We investigate the surface electronic state by examining the amplitude and phase of THz wave emitted from the sample upon the radiation of near-infrared laser pulse. A schematic of an optical configuration near the sample is shown in Fig. 1(a). Among several THz generation mechanisms, we consider the optical rectification and the laser-induced surge current as a primary source for BSTS^16^. Particularly in the latter case, the emitted THz wave can provide important information about the charge dynamics as well as about the surface band bending. Laser-induced surge current arises in terms of two representative mechanisms, i.e., photo-Dember effect and the band-bending-induced charge acceleration, as depicted in Fig. 1(b,c), respectively. In the case of photo-Dember effect, a transient dipole is induced by a diffusion of photo-induced carriers, and hence the polarity of THz wave is determined by a mobility difference between electron and hole^17^. In the case of band-bending effect, on the other hand, the transient dipole is induced by the acceleration of electron and hole along the opposite directions by the built-in electric field in the space charge region, and hence the polarity of THz wave depends on the details of the band bending, i.e., whether it is upward or downward as depicted in Fig. 1(c)^18^. Therefore, by investigating the amplitude and phase of emitted THz wave, we can obtain information about the electrodynamics (particularly about the charge carrier mobility) or about the band bending in the space charge region^17^,^18^.

### Evolution of emitted THz waves after the sample cleavage

**Figure 2:**
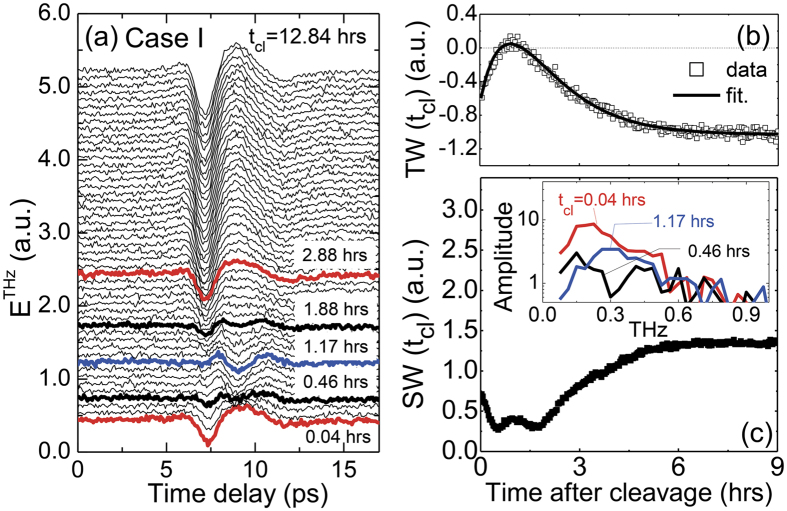
(**a**) Time(*t*)-domain electric-field profiles of emitted THz waves *E*^THz^(*t*) from Bi_1.5_Sb_0.5_Te_1.7_Se_1.3_ displayed in the chronological order after its cleavage in air (Case I). Five *E*^THz^(*t*) are highlighted, and they are obtained at *t*_cl_ = 0.04, 0.46, 1.17, 1.88, and 2.88 hours after the cleavage of the sample in an ambient air. (**b**) Integrated *E*^THz^(*t*) between *t* = 4.5 ps and 7.8 ps given as a function of *t*_cl_. (**c**) Integrated THz wave spectrum *Ẽ*^THz^(ω) between ω/2π = 0.04 THz and 0.79 THz plotted as a function of *t*_cl_. Note that both values in (**b**,**c**) are normalized by the values at *t*_cl_ = 9 hours. Inset shows three representative *Ẽ*^THz^(ω) obtained from the Fourier transform of *E*^THz^(*t*).

Figure 2(a) shows time(*t*)-domain electric-field profiles of THz waves *E*^THz^(*t*) emitted from BSTS obtained after the cleavage of the sample in an ambient air (Case I) with an oxygen content of 20.5% and a relative humidity of about 33%. Each measurement of *E*^THz^(*t*) took about two minutes, and it has been monitored as a function of time after the cleavage (*t*_cl_) up to about 14 hours. Just after the cleavage, i.e., *t*_cl_ ~ 0.04 hours (red one in the bottom), the wavefront of the THz wave shows a negative sign of the electric field in a temporal range between 4.5 and 7.8 ps. As time goes on after the cleavage, i.e., with an increase of *t*_cl_, this feature becomes weaker, and almost smeared out at *t*_cl_ = 0.46 hours (thick black line in the bottom side). Then *E*^THz^(*t*) develops to have an another distinct waveform (blue line) at *t*_cl_ = 1.17 hours which looks different from that of the initial THz wave. After that, the waveform is restored to the initial one, and then has a deep valley at the wavefront in the saturation limit, e.g., at *t*_cl_ = 13.7 hours. It should be noted that *E*^THz^(*t*) displays an isotropic azimuth dependence at *t*_cl_ > 13.7 hours (as demonstrated in Supplementary information), and hence we exclude the optical rectification from the candidates of possible THz generation mechanisms. Instead, we consider the surge current as a dominant THz emission mechanism at *t*_cl_ > 13.7 hours as well as *t*_cl_ ~ 0 hours when *E*^THz^(*t*) has a similar waveform. Therefore, the time-dependent evolution of the THz waveform can provide us with valuable information of the photocarrier dynamics near the sample surface.

We examine an evolution of the emitted THz wave in more detail by integrating the THz electric field *E*^THz^(*t*). When the THz wave is generated by the surge current mechanism, the THz electric field should be proportional to the time-derivative of the surge current *J*(*t*), i.e., 
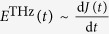
19. Therefore, the temporal integration of *E*^THz^(*t*) can provide information about the transient photocurrent along the surface normal direction^19^,^20^. To examine the initial charge dynamics, in particular, we integrate the wavefront side, i.e., *E*^THz^(*t*) in the temporal range between 4.5 ps and 7.8 ps. This temporal weight *TW* of *E*^THz^(*t*) is normalized with the value at *t*_cl_ = 9 hours, and plotted as a function of *t*_cl_ in Fig. 2(b). *TW* has a negative value just after the cleavage which corresponds to the dip structure in *E*^THz^(*t*). As time goes on, *TW* approaches a zero level, and crosses over it to have a small but positive value around 1.17 hours. Then it returns back to have a negative value, and is finally saturated from about 6 hours after the cleavage.

We can find a similar behavior of the THz emission response from the spectral weight *SW* analysis. Electric field amplitude of the THz wave in a frequency domain, i.e., *Ẽ*^THz^(ω) is obtained by the Fourier transform of *E*^THz^(*t*), and the inset of Fig. 2(c) displays *Ẽ*^THz^(ω) at *t*_cl_ = 0.04, 0.46 and 1.17 hours. We integrate these spectra from 0.38 THz to 0.79 THz, and display the *SW* values as a function of *t*_cl_ in Fig. 2(c). The time(*t*_cl_)-dependent *SW* shows a similar behavior with *TW*; as time goes on, *SW* decreases first, and it increases until it saturates at about *t*_cl_ = 6 hours. It is interesting to note that it has a local maximum around *t*_cl_ = 1.17 hours between two minima. Actually, these minimum *SW*s are obtained when *TW* crosses the zero level before and after having the positive maximum at *t*_cl_ = 1.17 hours. These clearly demonstrate that the THz waveform experiences a symmetric time-dependent changes with respect to *t*_cl_ = 1.17 hours, and there exist two competing contributions to the observed THz emission signal as a function of *t*_cl_.

### Possible flat band-bending at the BSTS surface

**Figure 3:**
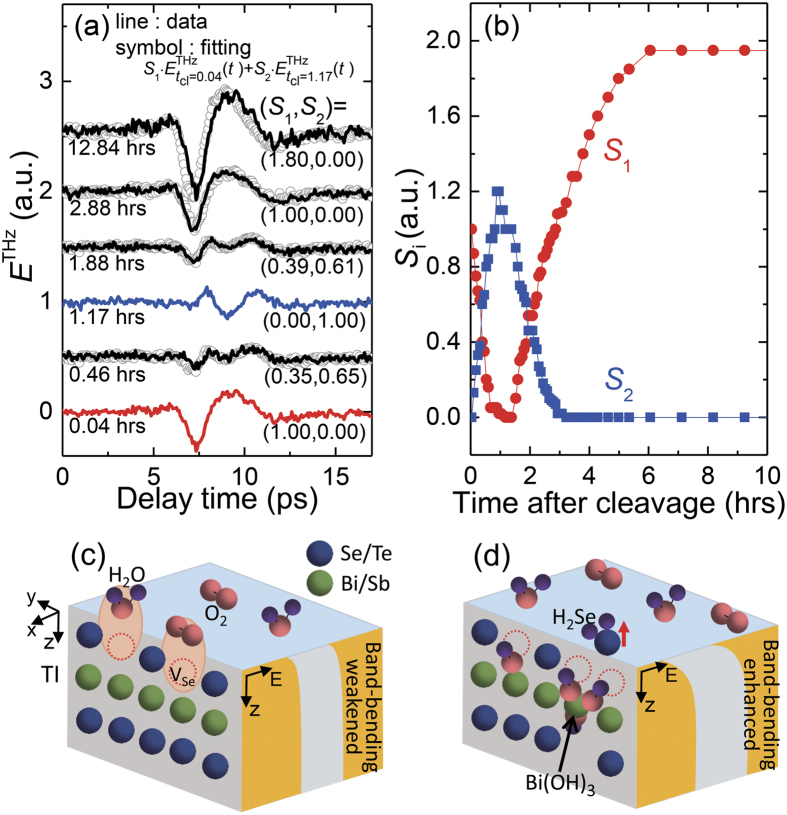
(**a**,**b**) Fitting analysis of *E*^THz^(*t*) with a linear combination of two waves at *t*_cl_ = 0.04 hours and 1.17 hours. Fitting curves of *E*^THz^(*t*) are shown with symbols in (**a**). The weighting factors *S*_1_ and *S*_2_ for each contribution are plotted in (**b**). (**c**,**d**) Cartoons describing interaction between Bi_1.5_Sb_0.5_Te_1.7_Se_1.3_ and atmospheric molecules, i.e., H_2_O and O_2_. Just after the sample cleavage (**c**), O_2_ and H_2_O molecules are adsorbed at the surface, and they act as electron acceptors. Then the chemical reaction occurs between the sample and the molecules (**d**), and it leads to the n-type doping in the sample surface.

It is important to point out that the existence of two different contributions to the THz signal can be clearly resolved by closely examining THz waveforms in the intermediate states. Just after the cleavage and also at the saturation stage, *E*^THz^(*t*) has a well-defined single-cycle waveform. On the other hand, at *t*_cl_ = 1.17 hours when *TW* and *SW* have a global and local maximum, respectively, we suppose that *E*^THz^(*t*) has another distinct form with a one-and-half cycle oscillation. Interestingly, the THz waveform in between, i.e., at 0.04 < *t*_cl_ < 1.17 hours, is not well characterized by any of these two waveforms. Instead, it looks that both contributions coexist. For example, *E*^THz^(*t*) at *t*_cl_ = 0.46 and 1.88 hours has a dip at the wavefront and additional double peaks following it which resemble the characteristic features of *E*^THz^(*t*) at *t*_cl_ = 0.04 and *t*_cl_ = 1.17 hours, respectively. Furthermore, whereas *Ẽ*^THz^(ω) at *t*_cl_ = 0.04 and *t*_cl_ = 1.17 hours have a single maximum (inset of Fig. 2(c)), *Ẽ*^THz^(ω) at *t*_cl_ = 0.46 hours has a dip around 0.3 THz between two maxima which is a typical spectral feature appearing when there are two consecutive pulses with a finite time interval. Actually, experimental results of *E*^THz^(*t*) can be reproduced quite well by a linear combination of aforementioned distinct waveforms as 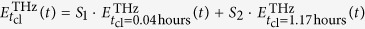. The fitting curves based on this equation are plotted as solid lines on top of the experimental data in Fig. 3(a). Obtained coefficients *S*_1_ and *S*_2_ are displayed as a function of *t*_cl_ in Fig. 3(b). These values take completely opposite time-dependent changes at *t*_cl_ < 3 hours, and then only *S*_1_ keeps increasing until it becomes saturated at about *t*_cl_ = 6 hours. These results demonstrate that there exist two different THz emission mechanisms, and they contribute to the THz response differently upon the evolution of the surface state as a function of time.

As a dominant THz emission mechanism in the saturation stage, we already excluded the optical rectification, and now consider the charge acceleration due to the downward band bending because of following reasons. When BSTS is exposed to the air, it is well accepted that the final surface state has the downward band bending^9^,^11^. Since the downward band bending is largely attributed to the extrinsic contributions, such as a selenium vacancy, chemical adsorption and reaction^7^,^8^,^9^,^21^, it is expected to have a formation of a larger number of charged scattering centers which will reduce the contribution of photo-Dember effect. Therefore, the increasing tendency of the THz emission amplitude before its saturation should be attributed to the larger contribution of the downward band bending. This leads to the assignment of the THz emission mechanism for the contribution *S*_1_ to be the charge acceleration due to the downward band bending. Therefore, the fact that the contribution of *S*_1_ becomes almost disappeared at about *t*_cl_ = 1.17 hours possibly indicates that the flat band bending can be achieved in the intermediate state during the maturation of the surface state in the atmospheric condition.

### Distinct surface-state evolution depending on environmental conditions

**Figure 4:**
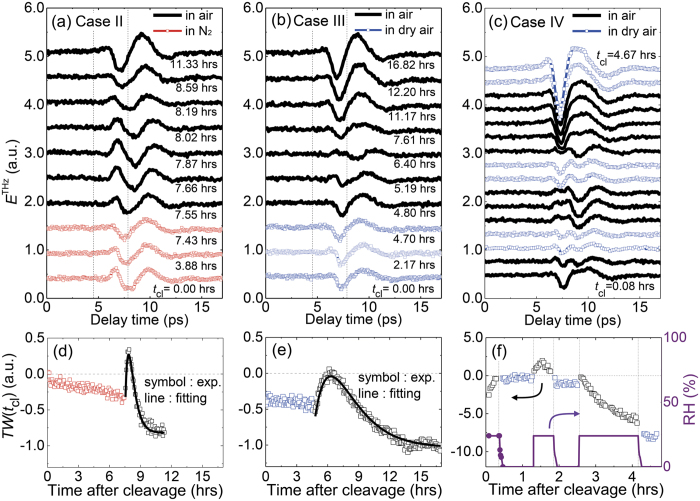
THz wave emission responses of BSTS after its cleaving in N_2_ atmosphere (Case II), in dry air (Case III), and in the ambient/dry air (Case IV). In Cases II and III, the sample is exposed to the ambient air after keeping the sample in the initial condition for several hours. In Case IV, the condition is consecutively switched between humid air with a relative humidity of 23% and dry air with the humidity less than 6%. Results obtained in the initial conditions are displayed with red and blue colors for the Case II and Case III, respectively, and the results obtained in the ambient air are displayed in black. Electric-field profiles of emitted THz waves *E*^THz^(*t*) are shown in (**a**–**c**). Integrated *E*^THz^(*t*) is plotted as a function of the elapsed time after the cleavage in (**d**–**f**). Solid lines in (**d**,**e**) are fitting curves using two exponential functions. The time-dependent relative humidity (RH) is displayed in (**f**) where a solid line is reproduced from the measured values shown with a symbol.

By monitoring time-dependent THz emission responses after the sample cleavage in the ambient air, we have demonstrated that the surface states of BSTS are strongly affected by the atmospheric conditions, and there can be a surface state condition close to the flat band bending during the maturation process. Now, we present distinct time-evolutions of the THz emission responses in different environmental conditions which reveal the effect of ambient gases on the surface electronic structure in more detail. As a Case II, we cleaved the sample in the N_2_ atmosphere with the oxygen content less than 0.3% and RH of about 2.0%, and kept the sample in this environment for about 7.5 hours before changing the condition to the ambient air environment as in Case I. After the cleavage in N_2_, there is a gradual variation of THz emission signal (Fig. 4(a,d)). Such tiny change can be further minimized by cleaving the sample in the dry air. As a Case III, we cleaved the sample and kept it for about 4.7 hours in dry air with RH at a zero level, and then changed the environmental condition to the ambient air. As shown in Fig. 4(b,e), THz emission signals exhibit essentially no time-dependent change when the sample is cleaved in the dry air.

In both Cases of II and III, as the conditions are changed to the ambient air containing a relative humidity of about 33%, dramatic changes in *E*^THz^(*t*) occur, and their time-dependences are quite similar with the result for the Case I; all of them have lambda-like shapes of *TW*(*t*_cl_). In particular, the distinct results for Case III obtained in dry and ambient air clearly demonstrates the important role of H_2_O molecules in the evolution of the surface electronic state after the sample cleavage. Although overall behaviors in the ambient air appear similar for all the Cases, it should be noted that the time scales of such variations are given distinctly. We fit time profiles of *TW*(*t*_cl_) with two exponential functions as 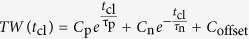, where *C*_offset_ is a constant offset level, τ_p_ and τ_n_ are relaxation times of the exponential function with positive and negative slopes, respectively, and *C*_p_ and *C*_n_ are corresponding weighting parameters. The fitting curves are shown with solid lines in each *TW*(*t*_cl_) plot, and the decay time (τ_p_, τ_n_) used for the fitting in Cases I, II, and III are given as (1.0, 1.2), (0.3, 0.5), and (1.8, 2.0), respectively, in the unit of hours. It should be noted that this process occurs the fastest for the Case II and the slowest for the Case III.

### Possibility to prolong the period of flat band-bending condition

Whereas we found a clear signature of the flat band bending during the maturation process of the surface state in the ambient air, we examined whether such an intriguing condition can be maintained by further controlling environmental conditions. Figure 4(c) displays time-dependent evolutions of THz emission responses of BSTS under the consecutive switches of environmental conditions between the ambient air containing RH of 23% and the dry air with RH level less than 6%. When the sample is in the humid air condition, the THz emission responses (indicated with black color) show strong time-dependent changes, and overall behaviors appear quite similar with the results demonstrated for the Cases I–III. This includes the global maximum in the time-dependent envelop of the integrated temporal weight *TW* observed at *t*_cl_ ~ 1.4 hours after the cleavage, and it corresponds possibly to the flat band-bending condition. When the condition is changed into the dry air, however, time-dependent change appears quite differently; as indicated with blue color, the time-dependent variations can be almost stopped before and after the flat band-bending condition. (The increase of the THz emission amplitude at the saturation stage at *t*_cl_ ~ 4.2 hours is attributed to the less atmospheric absorption of THz wave in the dry air condition). This clearly demonstrates that we can prolong the period of the flat band-bending condition before reaching the irreversible saturation stage by reducing the contributions of H_2_O molecules in the air.

### Chemical reactions and surface state evolution

From now on, we discuss a possible mechanism of the surface state evolution related to the band bending after the cleavage of the BSTS crystal. For topological insulators, it is usually not straightforward to determine the direction of the band bending as the surface state of topological insulators is very dispersive, and hence the Fermi level pinning to the high-density surface state is not simply applicable^22^,^23^. In this work, however, we address this issue by examining the THz emission responses in detail. As discussed in the above, the THz emission at the saturation state is attributed to the surge current driven by the downward band bending. Since *E*^THz^(*t*) obtained just after the sample cleavage looks essentially the same with that at the saturation state (Fig. 2(a)), it is reasonable to consider that the downward bend banding occurs also just after the sample cleavage. Actually, this interpretation is supported by the comparison with the result for InAs. The observed THz waveform (Fig. 2(a)) shows an opposite phase to that of InAs (Supplementary information) where the THz emission is attributed to the photo-Dember effect. As the electron mobility is larger than the hole for InAs, the corresponding THz waveform is expected to have the same phase with the THz wave generated by the upward band bending (See Fig. 1(b,c)). Therefore, the opposite phase for BSTS with respect to that for InAs also suggests the downward band bending just after the sample cleavage. *E*^THz^(*t*) for Case II just after the cleavage exhibits a waveform different from the other cases, but it resembles the waveform for Case I with a minimal downward band bending. Hence, this implies that the downward band bending would be much weakened in this case. Nevertheless, *E*^THz^(*t*) after a few hours recovers the waveform corresponding to the downward band bending. This is attributed to the formation of the negatively charged Se vacancies V_Se_ near the surface after the cleavage even in the N_2_ atmosphere^21^,^24^, and the accumulation of more electrons at the surface. This assures again our interpretation of the initial state (except for the Case II) to have the downward band bending.

When the sample is cleaved in the ambient air, the fresh surface state will be strongly affected also by an adsorption of the environmental ions, such as N_2_, O_2_, CO_2_, CO, and H_2_O, and even their chemical reactions with the sample^7^,^8^,^25^,^26^,^27^,^28^. Among these molecules, we take account of the contributions of O_2_ and H_2_O mainly, but discard the effects of other molecules by considering the inert nature of N_2_ and tiny contents of CO_2_ and CO (about 400 ppm and 0.01 ppm, respectively) in the air (See Supplementary information for the details). Since O_2_ has a strong electron affinity, they are adsorbed to the TI surface to attract excess electrons accumulated at the surface (Fig. 3(c))^7^,^24^. When the sample is cleaved in the dry air condition (Case III), the THz emission response shows almost no time-dependence which is attributed to the balance between the surface charge accumulation originating from the V_Se_ and the electron depletion by O_2_ molecules. Such tendency to reduce the electron accumulation can be further enforced by H_2_O which has a large electron affinity and electric dipole moment (see Supplementary information)^21^. After the cleavage in the ambient air, hence the adsorption of both O_2_ and H_2_O occurs, and it tends to neutralize the surface charge state which was originally electron-accumulated. During this process, the number of charged particles near the surface is reduced, and accordingly the built-in electric field becomes smaller as indicated in the right bottom surface of Fig. 3(c). Therefore, *E*^THz^(*t*) generated from the built-in field should be reduced. These behaviors could explain the history of *E*^THz^(*t*) in the initial stage after the cleavage in the ambient air, i.e., *t*_cl_ < 1.17 hours for Case I. With a further elapse of time, actual chemical reactions can occur between BSTS and molecules in air. For example, H_2_O is dissociated into H and OH in the existence of O_2_ gas, and accelerates the formation of V_Se_ by forming a H_2_Se gas and also by leaving 2Bi(OH)_3_ in the sample as depicted in Fig. 3(d)^8^,^21^. This process contributes to the more charge accumulation near the surface which is accompanied by the enhancement of the band bending as indicated in the right bottom surface of Fig. 3(d). Therefore, *E*^THz^(*t*) generated from the built-in field should be enhanced, and this explains the history of *E*^THz^(*t*) after *t*_cl_ = 1.17 hours for Case I. It is worth to emphasize that the driving mechanisms for the charge accumulation and neutralization are competing with each other in BSTS, and there can be a moment to have an intermediate state with a negligible band bending after the sample cleavage.

As demonstrated for Cases I–III, the surface state evolution in the ambient air occurs with different time scales depending on the treatments done before switching the condition into the ambient air. To account for the difference in the decay time during the maturation process, we consider the effective amount of V_Se_ at the moment when the atmospheric condition is changed into the air. For the case II, V_Se_ keeps being formed in the N_2_ atmosphere, and hence there is an enough chance of the interaction between V_Se_ and ambient molecular components which results in a fast adsorption of the molecules and accordingly the fast relaxation process. For the case III, on the other hand, the naturally formed V_Se_ is already compensated by the adsorption of O_2_ in a dry air condition. Therefore, it takes a longer time for the system to be relaxed into the final state even with an additional contribution of H_2_O molecules. It is worth to note that the surface electronic state evolution of BSTS for all three cases investigated shows distinct behaviors when it is compared to that of Bi_2_Se_3_ which exhibits faster and monotonic variations of the THz emission^14^ and optical second harmonic generation responses^24^ after the sample cleavage. As one of possible reasons of these differences, we consider the suppression of the V_Se_ formation for BSTS where the Se ions are trapped at the center of the quintuple layer with a strong chemical bonding with Bi ions^29^. This leads to a relatively slow progress of the surface state stabilization, and hence provides a chance to trace such evolutions in more detail.

In summary, we investigated a time-dependent evolution of the surface state of the Bi_1.5_Sb_0.5_Te_1.7_Se_1.3_ single crystal after the sample cleavage by examining THz light emitted from the sample surface upon the illumination of the near-infrared femtosecond laser pulses. We observed strong variations of THz emission responses as a function of time after the sample cleavage which strongly depend on environmental conditions. In particular, we observed a clear signature of the flat band bending condition of the surface electronic structure in the course of the interaction between Bi_1.5_Sb_0.5_Te_1.7_Se_1.3_ and environmental molecules. Furthermore, we could demonstrate that the evolution of the surface electronic state can be accelerated, decelerated, and even stopped by controlling the atmospheric conditions. This assures that the surface decoration can work as an efficient way to tune the Fermi level of Bi_1.5_Sb_0.5_Te_1.7_Se_1.3_ with respect to the surface Dirac state. Since such intriguing controllability could be achieved in the atmospheric condition at room temperature, it will provide a novel chance to exploit the topological surface state for the further practical researches, such as the spin photocurrent generation, with a minor contribution from the residual bulk carriers in the bulk-insulating Bi_1.5_Sb_0.5_Te_1.7_Se_1.3_.

## Methods

A high-quality single crystal of Bi_1.5_Sb_0.5_Te_1.7_Se_1.3_ (BSTS) was grown using the self-flux method with stoichiometric chunks, and it has an optically flat surface along the (111) direction with a lateral size of about 2 mm in diameter. Terahertz (THz) emission experiments were done by using a conventional THz time-domain spectroscopy setup. Laser pulse has a central wavelength of 800 nm and pulse width of about 100 fs with a repetition rate of 80 MHz, and is incident onto the sample with an average power of 230 mW at the incidence angle of 45°. As the laser beam size is large with a full width at half maximum of about 1.2 mm, the emitted THz wave follows a specular reflection of the laser beam^20^. Hence we collect the THz beam along the angle of 45° which is guided by a pair of parabolic mirrors and detected by using a photo-conductive antenna.

The O_2_ content and the relative humidity (RH) are controlled by flushing the N_2_ gas and the dry air, and they are monitored in real time by using oxygen analyzer (Greisinger) and hygrometer (Center). The ambient air conditions for Cases I, II, and III contain O_2_ molecules of 20.5% and RH of 33%. The O_2_ content and RH in the N_2_ atmosphere for Case II are 0.3% and 2.0%, respectively, and the corresponding values in the dry air for Case III are 20.5% and 0.0%, respectively. Note that the maximum fluctuation of RH is about ±2.5% of each measured value. In Case IV, the condition is consecutively switched between a humid air with RH 23% and dry air with RH less than 6% whereas the O_2_ content is maintained at 20.5%. Upon flushing the dry air, RH drops down to 6% in less than two minutes when the first measurement is launched. The other molecules, such as CO_2_ and CO, are not controlled intentionally, and expected to have usual volume fractions of about 400 ppm and 0.1 ppm, respectively, in the ambient air.

## Additional Information

**How to cite this article**: Park, S.H. *et al*. Possible flat band bending of the Bi_1.5_Sb_0.5_Te_1.7_Se_1.3_ crystal cleaved in an ambient air probed by terahertz emission spectroscopy. *Sci. Rep.* **6**, 36343; doi: 10.1038/srep36343 (2016).

**Publisher’s note:** Springer Nature remains neutral with regard to jurisdictional claims in published maps and institutional affiliations.

## Supplementary Material

Supplementary Information
