# Supplementary material for: Possible flat band bending of the Bi1.5Sb0.5Te1.7Se1.3 crystal cleaved in an ambient air probed by terahertz emission spectroscopy
Source: Sci Rep. 2016 Nov 2;6:36343. doi: 10.1038/srep36343 (PMC5090861; doi:10.1038/srep36343)
Supplement: Supplementary Information [file srep36343-s1.pdf]

Supplementary information

Possible flat band bending in the cleaved $Bi_{1.5}Sb_{0.5}Te_{1.7}Se_{1.3}$ crystal in an ambient air probed by terahertz emission spectroscopy

Soon-Hee Park[1], Sun Young Hamh[1], Joonbum Park[2], Jun Sung Kim[2], and Jong Seok Lee[1,*]

[1] Department of Physics and Photon Science, Gwangju Institute of Science and Technology, Gwangju 500-712, Republic of Korea
[2] Department of Physics, Pohang University of Science and Technology, Pohang 790-784, Republic of Korea
*jsl@gist.ac.k

Characterization of bulk properties

We characterized bulk properties of a single crystalline $Bi_{1.5}Sb_{0.5}Te_{1.7}Se_{1.3}$ (BSTS) based on the charge transport and optical measurements. Figure S1(a) shows a temperature (T) dependent DC resistivity (ρ_{xx}) which exhibits a typical insulating behavior at T>50 K. Fitting with an Arrhenius-type function (red curve in the inset of Fig. S1(a)) gives an activation energy of about 30 meV. At T<50 K,

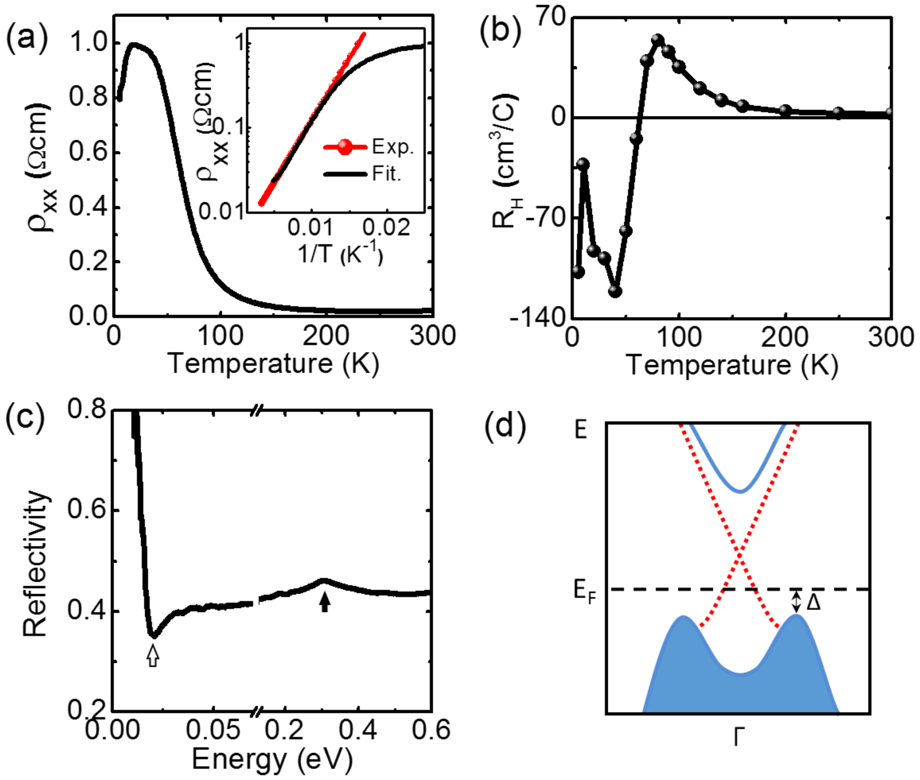

Supplementary Figure S1. Bulk characteristics for $Bi_{1.5}Sb_{0.5}Te_{1.7}Se_{1.3}$. (a) Temperature-dependent DC-resistivity. Symbols in the inset shows an Arrhenius fitting result in the temperature range from 80 K to 200 K. (b) Temperature-dependent Hall resistivity showing a sign change around 65 K. (c) Infrared reflectivity spectrum where the effective plasma frequency (~190 cm^{-1}) and Burstein frequency (~2,500 cm^{-1}) are indicated by arrows. (d) Schematic of the electronic structure near the Γ point. The excitation energy Δ is defined as an energy difference between the impurity level and the uppermost filled level of the valence band.

ρ_{xx} is saturated and shows a reduction with a further decrease of T which is attributed to the bulk insulating and surface metallic state of BSTS. The Hall resistance R_H shown in Fig. S1(b) undergoes a strong T-dependence; it has a positive value at room temperature and a large negative value at T<50 K which are attributed to the change of major charge carrier upon the temperature variation. Note that these behaviors are consistent with the recent report by Taskin *et al.*[1]. A reflectivity spectrum in the infrared region (Fig. S1(c)) exhibits clear signatures of a free carrier response and the gap excitation which appear as a reflectivity edge at $\omega_p^*/2\pi$~190 cm^{-1} and a hump structure at $\omega_B/2\pi$~2500 cm^{-1}, respectively. Using the carrier density (~2.2×10^{18} cm^{-3}) estimated from R_H and the plasma frequency ω_p^* (and also with the dielectric constant ϵ_∞~24 just below the gap energy), we determine the effective mass of the free carriers as $0.31m_0$ (m_0: bare electron mass), which is in good agreement with previous results[2]. Based on these results, we sketch the electronic structure of BSTS in Fig. S1(d). Since the activation energy obtained from ρ_{xx} is much lower than the optical gap, we introduce the impurity band inside of the band gap. As it is located near the valence band maximum, it also explains the temperature-dependent change of the majority carrier type[1]. Therefore, BSTS is a bulk-insulating TI with the Fermi energy E_F located in the energy band gap.

Discussion about the possible THz generation mechanism

We check the possibility of the optical rectification as a possibility of THz generation mechanism of BSTS by examining azimuth-dependent THz emission responses. Figure S2 displays time(t)-domain electric field profiles of emitted THz waves obtained with a full variation of the sample azimuth ϕ. Note that the results are obtained long after the sample cleavage. Here, both incident laser pulses and emitted THz waves are set to be p-polarized. Actually, THz light emitted through the optical rectification should reflect the symmetry property of the sample surface, i.e., 3m point group. Hence, the peak-to-peak amplitude of the emitted THz wave $E^{THz}_{peak\text{-}peak}$ would have a three-fold or six-fold symmetry in its azimuth dependences as demonstrated in the second harmonic generation[3]. As displayed in Fig. S2(b), the observed azimuth-dependence is fully isotropic, and hence we exclude the nonlinear optical rectification from the possible candidates of THz generation mechanisms in BSTS.

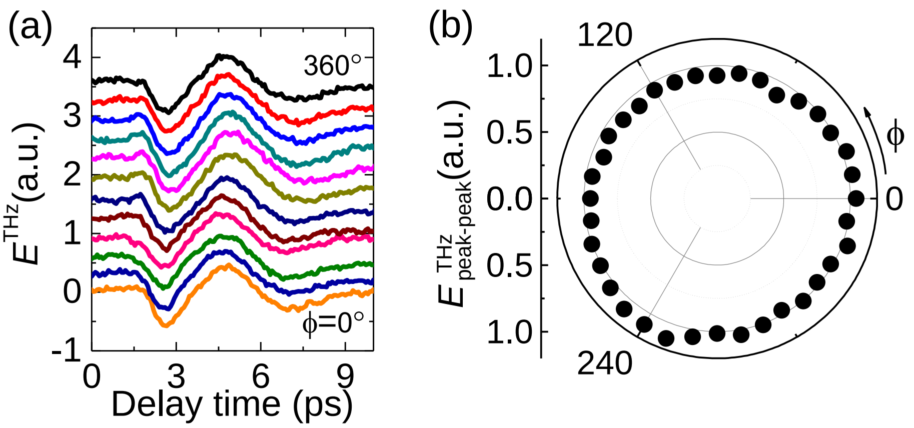

Supplementary Figure S2. Azimuthal angle (ϕ) dependent THz waves emitted from $Bi_{1.5}Sb_{0.5}Te_{1.7}Se_{1.3}$. (a) shows time-domain electric-field profiles of $E^{THz}(t)$ with varying ϕ by 15 degrees. (b) Polar plot for azimuth-dependent peak-to-peak amplitude of E^{THz}.

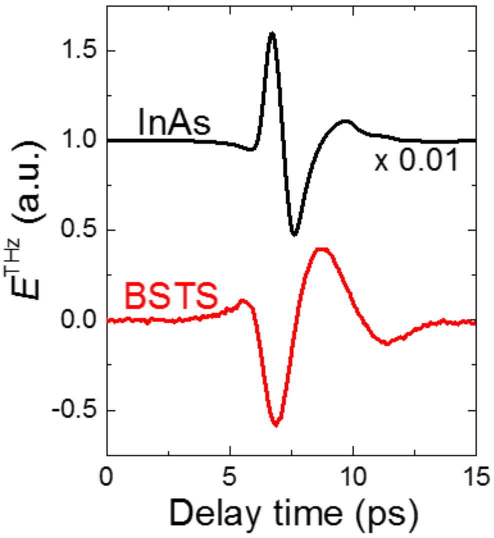

Supplementary Figure S3. Comparison between E^{THz}(t) from InAs and $Bi_{1.5}Sb_{0.5}Te_{1.7}Se_{1.3}$ (BSTS). Here, the result for BSTS is obtained just after the sample cleavage in the ambient air (Case I).

In the main text, we argue that the band bending-induced charge acceleration is the primary origin of the THz emission just after the cleavage as well as in the saturation state. Then the comparison with the result for InAs can provide us with valuable information about the band bending direction. For InAs, a dominant THz emission mechanism is the photo-Dember effect due to the larger mobility of electrons, and hence the corresponding E^{THz}(t) should have the same phase of E^{THz}(t) arising from the upward band bending[4]. Figure S3, however, exhibits that E^{THz}(t) from InAs and $Bi_{1.5}Sb_{0.5}Te_{1.7}Se_{1.3}$ have opposite phases, and this clearly demonstrates the downward band bending for the BSTS compound just after the sample cleavage.

Adsorption probabilities for gas molecules in the ambient air

The adsorption process is usually determined by three factors. First, the coverage of an adsorbate molecule on the surface is proportional to the partial pressure of the corresponding gas molecule. Second, the van der Waals interaction between the adsorbate and the given surface is a determining factor of the physical adsorption probability, and it is proportional to the polarizability and dipole moment of the molecules. Third, the chemical reactivity of the gas molecule can be the other important factor which determines the chemical adsorption probability. It should be noted that the physisorption contributes to the surface coverage much weakly compared to the chemisorption particularly at relatively high temperature, but it can play an important role in the adsorption process as it may act as precursors to the chemisorption.

Table S1 displays such information for representative gas molecules contained in the ambient air. As a parameter to represent the chemical reactivity, we list the electron affinity and ionization energy of the molecules whereas the ionization energies for all the molecules are similarly large. For N_2, although it is a major gas molecule in the ambient air, it is unpolarized and chemically inert. Accordingly, the only attraction between the N_2 molecule and the surface arises from van der Waals

Gases	Partial pressure (ppmv)	Polarizability (Å^3)[9]	Dipole moment (debye)[10]	Electron affinity (eV)[11-16]	Ionization energy (eV)[17-19]
N_2	780,840	1.710	0.00	-2.2	15.6
O_2	209,460	1.562	0.00	1.10	12.1
Ar	9,340	1.664	0.00	-11.5	15.8
CO_2	400	2.507	0.00	-0.60	14.2
CO	0.1	1.953	0.112	1.32	14.4
H_2O	8219[8]	1.501	1.85	1.3	12.6

Supplementary Table S1. Characteristic parameters of gas molecules in the ambient air related to the adsorption processes.

forces, and hence its adsorption to the surface is minimal. Actually, Brahlek *et al.* reported that the N_2 atmosphere has the same effect with the vacuum for Bi_2Se_3 thin films[5]. Note that the same analogy can be applied to the Ar gas. Although O_2 is also a non-polar molecule, we can consider its contribution to the adsorption since both the partial pressure and the electron affinity are large. CO_2 itself is expected to have the low adsorption rate as its partial pressure is small, the dipole moment is negligible, and also it is chemically stable. For CO, it may allow both physorption and chemisorption since it has relatively large values of polarizability, dipole moment, and electron affinity. Nevertheless, we do not consider its contribution seriously as it has a very tiny partial pressure in air. For reference, we mention that Yashina *et al.* and Bando *et al.* found no response of the carbonate-related species from the x-ray photoelectron spectroscopy experiment for Bi_2Te_3 within 8 hours and 24 hours[6,7], respectively, after the samples are cleaved in the ambient air.

In the ambient air, we have to consider the contribution of H_2O molecules as well; its partial pressure is about 8219 and 6476 ppmv for the relative humidity of 33 % and 26 % at room temperature, respectively. Also, it is a polar molecule. In particular, it can be easily dissociated into OH and O, and the dissorbate OH is a very reactive radical. Hence, H_2O can have a much higher effect on the adsorption process than other molecules. Consequently, we take account of O_2 and H_2O molecules as major adsorbates in the ambient air which can interact with the cleaved BSTS surface.

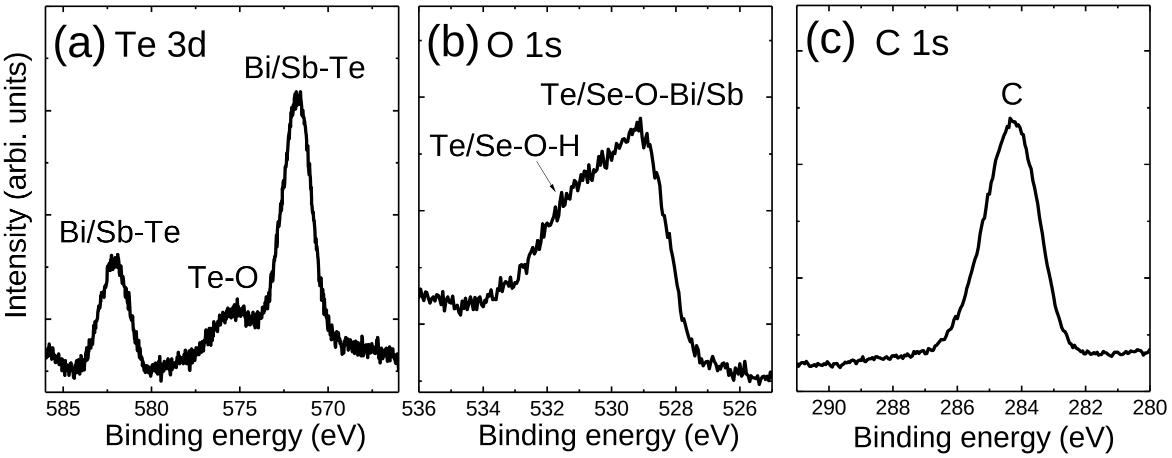

Supplementary Figure S4. Series of the Te 3d, O 1s, and C 1s core level spectra for BSTS surface in 30 hours after cleaving in the ambient air.

Figure S4 displays core level photoemission spectra of Te 3d, O 1s, and C 1s states for the BSTS sample which was taken at the beamline 4D of Pohang Light Source-II (PLS-II). Before the measurement under the ultrahigh vacuum condition (~8×10^{-10} Torr), the sample is cleaved and aged in the ambient air for 30 hours. The adsorption of the oxygen can be clearly observed for Te 3d peaks where a peak corresponding to Te-O bonding is observed together with the other peaks from the original chemical bonding[20]. Accordingly, O 1s peak appears broadly and asymmetrically, and shows a clear signature of the chemical adsorption of oxygen onto the BSTS surface[5]. C 1s peak also appears around 284.3 eV which corresponds to the response of the carbon itself[21]. Actually, we found no signature of the chemical bonding of the carbon with other ions in BSTS, and a weak spectral intensity of the carbon peak, i.e., less than 5 % of the total peak intensity in a wideband spectrum. We therefore conclude that the O and OH are adsorbed, but carbon has a minimal contribution to the adsorption.

References (Supplementary)

1. Taskin, A. A., Ren, Z., Sasaki, S., Segawa, K. & Ando Y., Observation of Dirac holes and electron in a topological insulator, *Phy. Rev. Lett.* **107**, 016801 (2011).
2. Tang, C. S. *et al.*, Terahertz conductivity of topological surface states in $Bi_{1.5}Sb_{0.5}Te_{1.8}Se_{1.2}$, *Sci. Rep.* **3**, 3513 (2013).
3. Hsieh, D. *et al.*, Nonlinear optical probe of tunable surface electrons on topological insulator, *Phys. Rev. Lett*. **106**, 057401 (2011).
4. Liu, K., Xu, J.-Z., Yuan, T. & Zhang, X.-C., Terahertz radiation from InAs induced by carrier diffusion and drift, *Phys. Rev. B* **73**, 155330 (2006).
5. Brahlek, M. *et al.*, surface versus bulk state in topological insulator Bi_2Se_3 under environmental disorder, *Appl. Phys. Lett.* **99**, 012109 (2011).
6. Yashina, L. V. *et al.*, Negligible surface reactivity of topological insulators Bi_2Se_3 and Bi_2Te_3 towards oxygen and water, *ACSNano* **7**, 5181-5191 (2013).
7. Bando, H. *et al.*, The time-dependent process of oxidation of the surface of Bi_2Te_3 studied by x-ray photoelectron spectroscopy, *J. phys. Condens. Matter* **12**, 5607-5616 (2000).
8. The value corresponds to the partial pressure for the relative humidity 33 % at 294 K.
9. Terry, N. O., Cann, N. M., Cooper, G. & Brion, C. E., Absolute scale determination for photoabsorption spectra and the calculation of molecular properties using dipole sum-rules, *Chem. Phys.* **223**, 59-98 (1997).
10. Nelson, R. D. Jr., Lide, D. R. Jr. & Maryott, A. A., *Selected values of electronic dipole moments for molecules in the gas phase* (ed. Astin, A. V.) 13, 15 (National standard reference data series-National bureau of standards 10, 1967).
11. Wulfsberg, G., *Inorganic chemistry* (ed. Stiefel, J.) 521 (University Science Books, 2000).
12. Stockdale, J. A. D., Compton, R. N., Hurst, G. S. & Reinhardt, P. W., Collisions of monoenergetic electrons with NO_2: Possible lower limits to electron affinities of O_2 and NO, *J. Chem. Phys.* **50**, 2176 (1969).
13. Andersen, T., Atomic negative ions: Structure, dynamics and collisions, *Physics Reports* **394**, 157–313. (2004).
14. Knapp, M., Echt, O., Kreisle, D., Märk, T. D. & Recknagel, E., Formation of long-lived CO_2^-, N^2O^-, and their dimer anions, by electron attachment to van der Waals clusters, *Chem. Phys. Lett.* **126**, 225 (1986).
15. Refaey, K. M. A. & Franklin, J. L., Endoergic ion-molecule-collision processes of negative ions. III. Collisions of I^- on O_2, CO, and CO_2, *Int. J. Mass Spectrom. Ion Phys.* **20**, 19 (1976).

16. Ballard, R. E., The electron affinity of water and the structure of the hydrated electron, *Chem. Phys. Lett.* **16**, 300 (1972).
17. Gáspár, R. & Nagy, Á., Ionization energies of the N_2, CO, CO_2, N_2O, C_2H_2 and SiH_4 molecules calculated by universal model potential, *Acta Physica Academiae Scientiarum Hungaricae* **50**, 359-365 (1982).
18. Dundas, D. & Rost, J. M., Molecular effects in the ionization of N_2, O_2, and F_2 by intense laser fields, *Phys. Rev. A* **71**, 013421 (2005).
19. Trofimov, A. B. & Schirmer, J., Molecular ionization energies and ground- and ionic-state properties using a non-Dyson electron propagator approach, *J. Chem. Phys*. **123**, 144115 (2005).
20. Thomas, C. R. *et al*., Surface Oxidation of $Bi_2(Te,Se)_3$ Topological Insulators Depends on Cleavage Accuracy, *Chem. Mater*., **28**, 35−39 (2016).
21. Moulder, J. F., *Handbook of X-ray photoelectron spectroscopy: A reference book of standard spectra for identification and interpretation of XPS data* (ed. Chastain, J.) 40-41 (Physical electronics, 1995).
